# ASSOCIATIONS BETWEEN COVID-19 RELATED STRESS, COVID-19 EXPERIENCES, AND MENTAL HEALTH RISKS IN OLDER PEOPLE

**DOI:** 10.1093/geroni/igac059.2993

**Published:** 2022-12-20

**Authors:** Tianyin Liu, Dara Kiu, Yi Leung, Wen Zhang, Wai-wai Kwok, Lesley Sze, Edwin Wong, Gloria H Y Wong, Terry Lum

**Affiliations:** The University of Hong Kong, Hong Kong, Hong Kong; The University of Hong Kong, Hong Kong, Hong Kong; The University of Hong Kong, Hong Kong, Hong Kong; The University of Hong Kong, Hong Kong, Hong Kong; The University of Hong Kong, Hong Kong, Hong Kong; The University of Hong Kong, Hong Kong, Hong Kong; The University of Hong Kong, Hong Kong, Hong Kong; The University of Hong Kong, Hong Kong, Hong Kong; The University of Hong Kong, Hong Kong, Hong Kong

## Abstract

There is increasing recognition of the need to understand the mechanism of psychological impact brought by COVID-19. The present research used the Delphi technique to develop a COVID-19-Related Stress Scale for older people in Hong Kong (CSS-old) (study one) and examined its associations with COVID-19 experiences and mental health risks (study two). In study one, 17 helping professionals and 20 service users co-developed an 8-item CSS-old through four rounds of Delphi. In study two, a cross-sectional telephone survey was conducted between April and June 2022 among 4,921 older people (age≥60) recruited through community centres. Respondents were assessed using Patient Health Questionnaire-2 (PHQ-2), Generalized Anxiety Disorder 2-item (GAD-2), and CSS-old; their experiences with COVID-19 (infection, close friend/family infection) and demographical information were collected. A three-factor solution of CSS-old was identified after dropping one item (X2(df) = 83.53(11), CFI=0.996, TLI=0.993, RMSEA=0.037): (1) disruption to routines; (2) fear of infecting families/friends; and (3) concern for the community’s health. Structural equation modelling analyses revealed that being female (B=0.45), having close friend/family infected (B=1.10) and having a pre-existing mental health condition (B=1.87) were positively associated with COVID-19-related stress. Infection of COVID-19 (BPHQ=0.22; BGAD=0.24) and a pre-existing mental health condition (BPHQ=0.71; BGAD=0.59) had direct associations with depressive and anxiety symptoms; COVID-19-related stress mediated the relationship between close friend/family infection with depressive (B=0.20) and anxiety symptoms (B=0.21, all p < 0.05). These results suggest that older people’s COVID-19-related stress is beyond infection of the disease, and different experiences with COVID-19 may increase depression and anxiety risks through different pathways.

